# Venogenic erectile dysfunction: diagnosis on computed tomography cavernosography and endovascular treatment using an anterograde access via deep dorsal penile vein

**DOI:** 10.1186/s42155-022-00283-5

**Published:** 2022-02-03

**Authors:** Hanno Hoppe, Nicolas Diehm

**Affiliations:** 1 SwissIntervention Microtherapy Center, Kornhausstrasse 8, 3013 Bern, Switzerland; 2 University of Bern, Bern, Switzerland; 3 Vascular Institute Central Switzerland, Aarau, Switzerland

**Keywords:** Erectile dysfunction, Impotence, Venous leak, Embolization

## Abstract

**Background:**

The underlying etiologies of erectile dysfunction may be manifold. Among them, vasculogenic etiologies are of increasing relevance and are not strictly limited to the elderly population. According to recent study, venogenic erectile dysfunction appears to be even more relevant than arteriogenic erectile dysfunction. Venogenic erectile dysfunction due to venous leakage causes insufficient penile blood retention. Proper diagnosis of venous leakage should include both color Doppler flow analysis and computed tomography cavernosography for adequate patient selection and treatment planning. Besides surgical ligation of penile draining veins, endovascular treatment methods may demonstrate more promising results. Especially endovascular embolization of venous leakage using an anterograde access via deep dorsal penile veins appears to be more beneficial for patients’ clinical outcome and awareness of this technique should be raised among endovascular interventionalists.

**Case presentation:**

A 47-year-old man was diagnosed with venogenic erectile dysfunction due to venous leakage on color Doppler flow analysis and computed tomography cavernosography. He did not respond to PDE-5-inhibitors. This patient demonstrated major venous leakage of paired deep dorsal penile veins via periprostatic veins and internal pudendal veins draining into both iliohypogastric veins. This patient’s venous leak was treated with endovascular embolization using an anterograde access via deep dorsal penile veins.

**Conclusion:**

This patient’s erectile dysfunction due to venous leakage, based on findings in color Doppler flow analysis and computed tomography cavernosography, was embolized using an anterograde access via deep dorsal penile veins as a minimally-invasive endovascular treatment option.

**Supplementary Information:**

The online version contains supplementary material available at 10.1186/s42155-022-00283-5.

## Background

Definition of erectile dysfunction is the recurring inability to achieve and maintain an erection for satisfactory sexual intercourse, which may result in relevant life impairment. Erectile dysfunction may be caused by various etiologies such as vasculogenic, endocrinologic, neurogenic, iatrogenic, psychogenic or rarely structural components. Not only in the elderly population, especially vasculogenic etiologies are increasingly relevant. Among them the incidence seems to be even higher for venogenic causes than for arteriogenic causes (Doppalapudi et al., 2019). Venogenic erectile dysfunction is due to insufficient penile blood retention during erection caused by venous leakage. For treatment of venous leakage surgical ligation of deep dorsal veins including potential collateral veins has been performed. However, surgical treatment is rather invasive and is usually performed under general anesthesia. Not very encouraging, long-term success rates of surgical ligation are reported to be 25% (Katzenwadel et al., 1993). Endovascular therapies with embolization of leaking veins have been reported including both retrograde and anterograde approaches with and without previous surgical exposure of deep dorsal penile veins (Spiliopoulos et al., 2013). However, the authors are confident that an anterograde approach with ultrasound-guided puncture of a deep dorsal penile vein is more beneficial for the patient in terms of clinical outcome. Therefore, we aim to describe and illustrate this technique to encourage other interventionalists to adapt to it. Furthermore, we intend to emphasize the need for pre-interventional work-up, especially computed tomography cavernosography, for adequate patient selection and treatment planning.

## Case presentation and diagnosis

A 47-year-old man was diagnosed with erectile dysfunction due to suspected venous leakage following an urological intervention not further specified 20 years ago. Patient especially complains about insufficient penile rigidity and early penile relaxation during sexual activity. Furthermore, he is a non-responder to PDE-5-inhibitors. Patient was assessed with an International Index of Erectile Function Questionnaire (IIEF-15) score of 43 indicating moderate erectile dysfunction.

### Color Doppler flow analysis

For patient work-up, initially color Doppler flow analysis was performed using direct pharmacological stimulation with an intra-cavernosal injection of 10 μg prostaglandin E1 resulting in penile tumescence grade E3 (considered not sufficient for sexual intercourse). At 15 min post injection (rigid phase), systolic flow rates of 50 cm/s (right cavernosal artery) and 47 cm/s (left cavernosal artery) (normal > 30 cm/s) and persistent end-diastolic velocities of 11 cm/s (right cavernosal artery) and 10.5 cm/s (left cavernosal artery) (normal < 5 cm/s) were found compatible with venous leakage (Varela et al., 2020).

### Computed tomography cavernosography

**Fig. 1:**
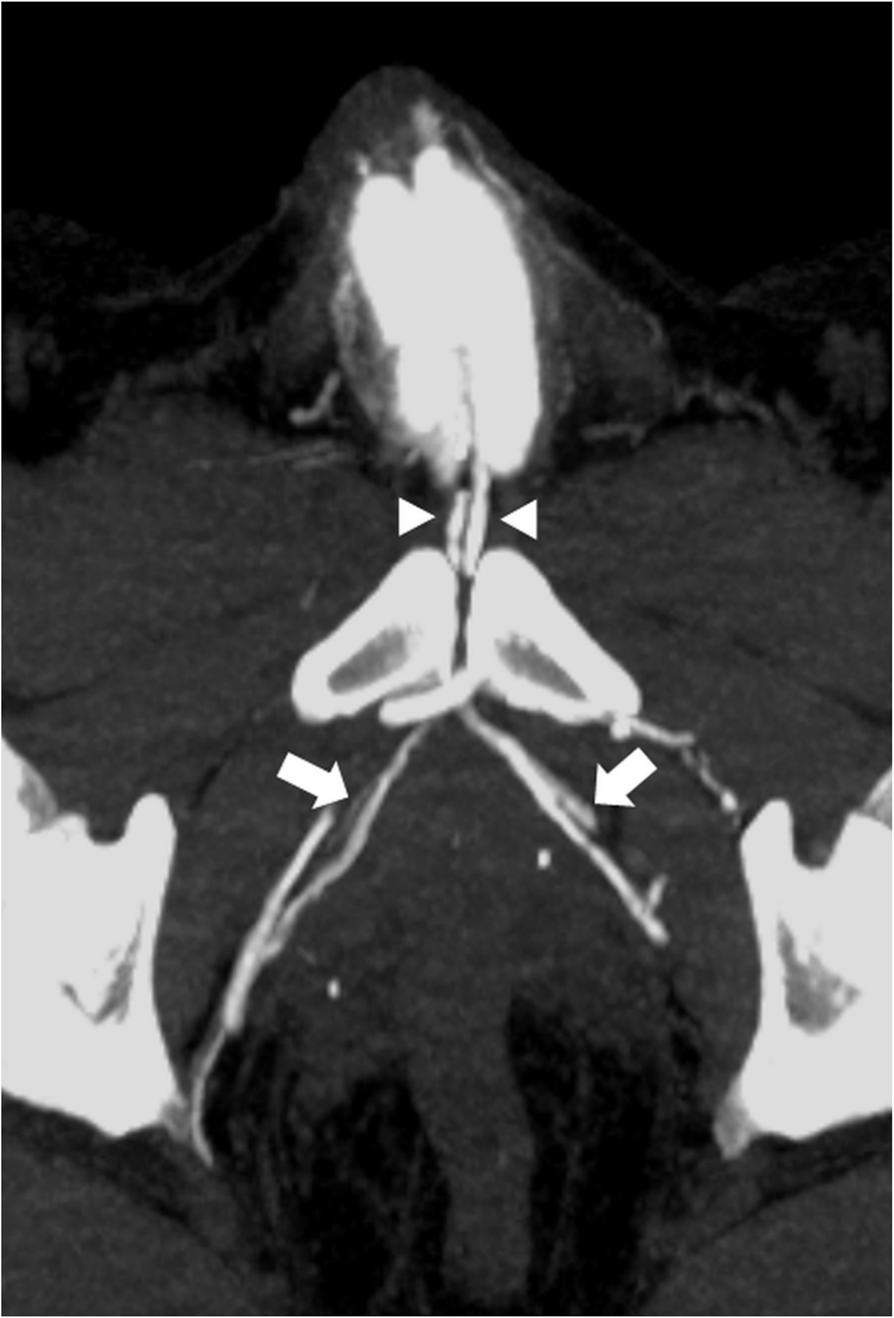
Contrast enhanced computed tomography cavernosography (maximum intensity projection) demonstrating major venous leakage from deep dorsal penile veins via bilateral periprostatic veins (arrows) draining into internal pudendal veins and bilateral iliohypogastric veins. Of interest, paired deep dorsal penile veins were found (arrowheads)

**Fig. 2:**
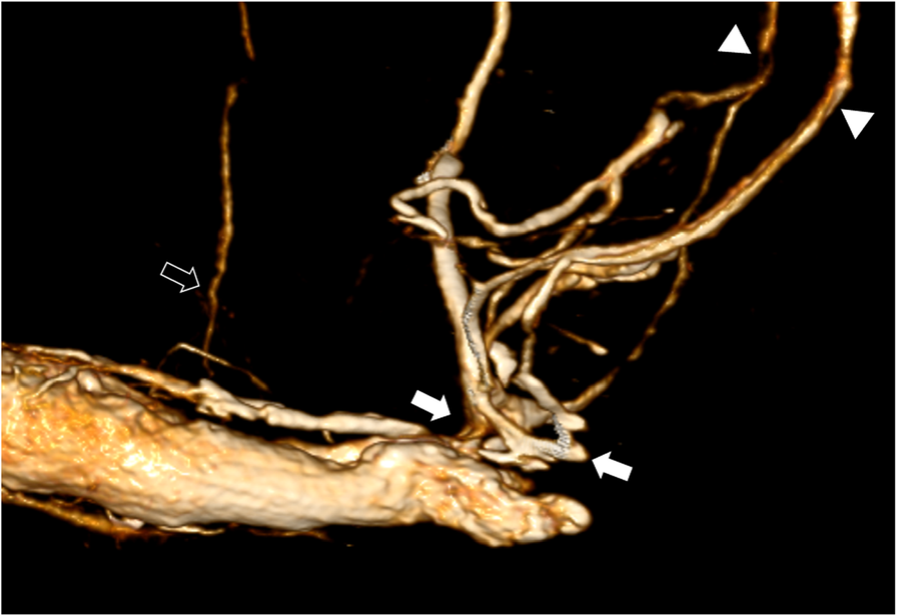
Contrast enhanced computed tomography cavernosography (volume rendering) demonstrating major venous leakage from deep dorsal penile veins via periprostatic veins (arrows) draining into internal pudendal veins and bilateral iliohypogastric veins (arrowheads). Besides there is minor venous leakage originating more peripherally via superficial inferior epigastric veins (open arrow)

For confirmation of diagnosis and anatomical depiction of venous leaks computed tomography cavernosography was performed post intra-cavernosal injection of 10 μg prostaglandin E1 resulting in penile tumescence grade E3. Fifteen minutes post injection a 23-G needle was inserted at the dorso-lateral side of the corpora cavernosum. Graduated injection of normal saline into corpora cavernosum at increasing flow rates up to a flow rate of 0.6 ml/s resulted in penile tumescence grade E4. Subsequently injection of 30 ml 50% saline-diluted non-ionic iodinated contrast medium (300 mg ml^− 1^) with an infusion velocity of 0.6. ml/s was performed. Computed tomography parameters were as follows: 64 × 0.625 mm collimation, gantry rotation time 0.75 s, time resolution 30 ms, pitch factor 0.984. Continuous scanning was performed under real time monitoring of contrast distribution up to the iliohypogastric veins extending from the upper brim of the true pelvis to the most distant level of the penis. The data constructive section thickness was 1 mm with a reconstruction increment of 1 mm for post-processing. For post-processing, multiplanar reformation using maximum intensity projection and volume rendering was applied. This patient demonstrated major venous leakage of paired deep dorsal penile veins via periprostatic veins and internal pudendal veins draining into both iliohypogastric veins (Fig. 1). Furthermore, a more peripheral minor venous leak was found with drainage to superficial inferior epigastric veins (Fig. 2).

## Endovascular treatment

**Fig. 3:**
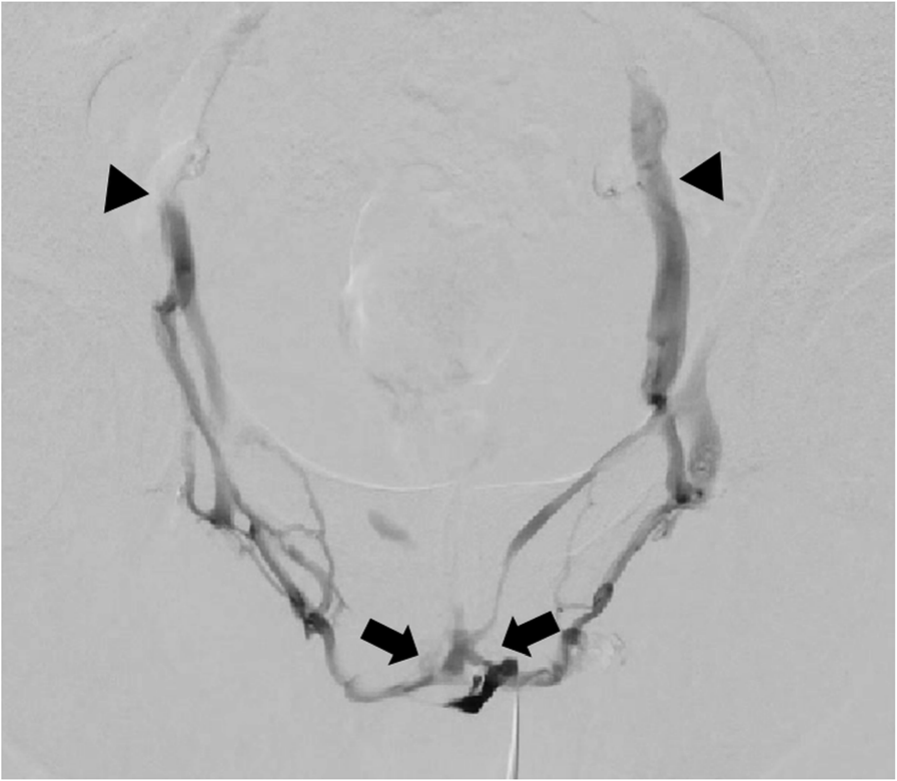
Venogram post anterograde access via deep dorsal penile vein confirming venous leakage via bilateral periprostatic veins (arrows) and internal pudendal veins draining into iliohypogastric veins (arrowheads)

**Fig. 4:**
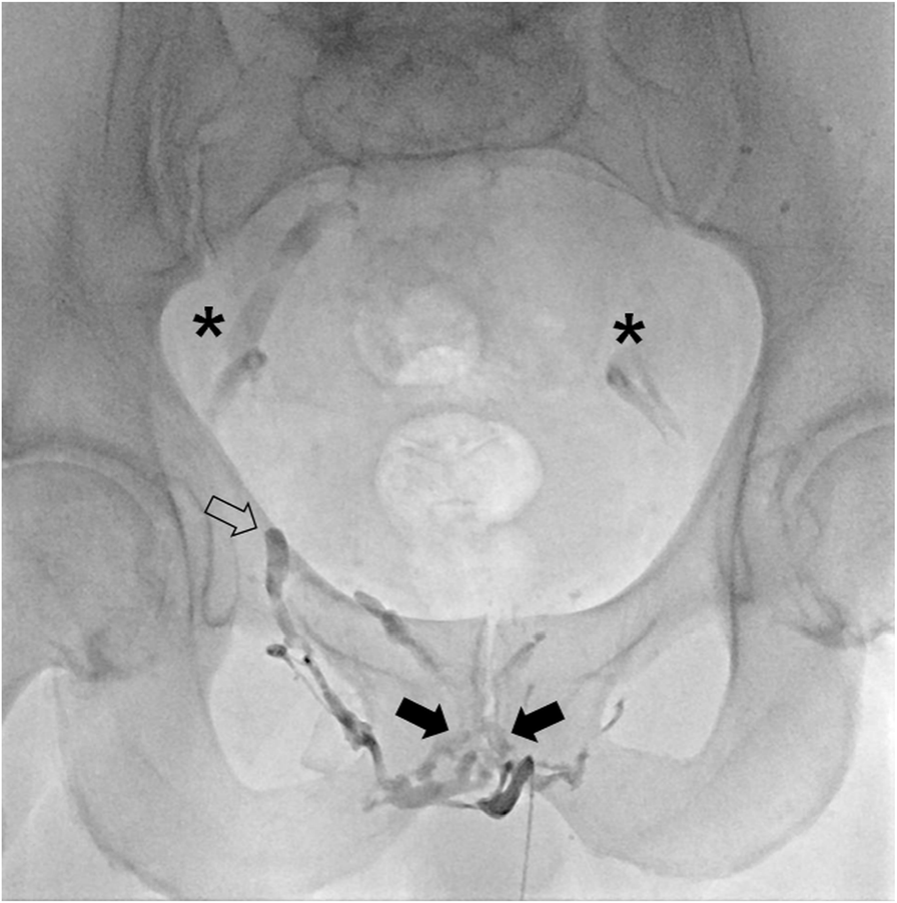
Radiographic image post venous leakage embolization using N-butyl-2-cyanoacrylate and ethiodized oil mixed in a 1:3 ratio. Note radiopaque embolization material within periprostatic veins (arrows) and internal pudendal vein (open arrow). There is residual contrast staining of both iliohypogastric veins post venogram (asterisk) as also demonstrated in the complementary movie file, not to be mistaken for embolization material. Complementary movie file demonstrating venous embolization performed with a slow but steady injection of embolization material under Valsalva maneuver and continuous fluoroscopic monitoring

The treatment strategy for this patient was embolization of major penile venous leakage. The patient was consented beforehand. The procedure was performed after direct pharmacological stimulation with an intra-cavernosal injection of 10 μg prostaglandin E1 was performed. Peri-interventional antiphlogistic and pain medications were administered. The patient was prepared and draped in the supine position. Following local subcutaneous administration of lidocaine 2% for local anesthesia and light sedation, an ultrasound-guide puncture of a penile deep dorsal vein was performed using a stiff 20-G micropuncture set with a 0.018-in. guide wire and 4-French introducer (Cook Inc., Bloomington, Indiana, U.S.A.). The stiff set appears to be advantageous compared to a regular floppier micropuncture set. The introducer was carefully advanced and positioned in close proximity to the radix penis and a diagnostic venogram was acquired confirming venous leakage via periprostatic veins and bilateral internal pudendal veins draining into both iliohypogastric veins (Fig. 3). Subsequently, all materials were flushed using 5% glucose solution to preserve catheter patency and prevent its inadvertent adhesion to the vessel wall. Afterwards venous embolization was performed with a slow but steady injection using N-butyl-2-cyanoacrylate (Histoacryl, Braun, Melsungen, Germany) and ethiodized oil (Lipiodol by Guerbet, Zurich, Switzerland) mixed in a 1:3 ratio under Valsalva maneuver and continuous fluoroscopic monitoring (Fig. 4). The injection was terminated in time prior to inadvertent distribution of embolization material to the iliohypogastric veins. The total amount of N-butyl cyanoacrylate used in this case was 3 ml. The more peripheral minor venous leak draining into superficial inferior epigastric veins was not treated at the time. Whenever necessary, these veins would rather qualify for percutaneous venous sclerotherapy than for embolization since reflux of embolization material into deep dorsal penile veins should be avoided since it may cause phlebitis.

## Clinical outcome

On 3-months follow-up this patient’s erectile dysfunction had improved as demonstrated on a semiquantitative improvement scale from 1 (maximum improvement) to 7 (no improvement) where patient had a score of 2 (almost maximum improvement). In addition, patient was assessed with an International Index of Erectile Function Questionnaire (IIEF-15) score of 57.

## Discussion and conclusion

In patients with erectile dysfunction due to venous leakage embolization is a minimally-invasive endovascular treatment option. Technical success rates are reported to be as high as 97% and complications rates are low (5%), including mainly minor complications whereas major complications such as pulmonary embolism are very rare (< 1%) (Doppalapudi et al., 2019). As reported in the literature, the average overall clinical success rate is 60% (range 22–100%) including various techniques and both partial and full responses (Doppalapudi et al., 2019; Fernandez Arjona et al., 2001; Rebonato et al., 2014; Peskircioglu et al., 2000). For full response, meaning sufficient erection to perform intercourse without additional need for supportive vasoactive medications, success rates rather tend to be within the lower range of the spectrum so far (Kutlu & Soylu, 2009). Durability of successful treatment was reported up to 22 months post embolization (Doppalapudi et al., 2019). However, small patient collectives on the one hand as well as inhomogeneous study protocols and various embolization techniques on the other hand may impede study reproducibility. Furthermore, long-term results are still pending. In conclusion, increasing future use of computed tomography cavernosography as routine approach in patients with suspected venous leak on color Doppler flow analysis for adequate patient selection and treatment planning as well as use of anterograde embolization techniques among endovascular interventionalists may contribute to patient health and ameliorated study results.

## Supplementary Information

**Additional file 1: Figure S1.** Computed tomography cavernosography with axial maximum intensity projection.

**Additional file 2: Figure S2.** Computed tomography cavernosography with three-dimensional volume rendering (quod vide complementary movie file including three dimensional maximum intensity projection).

**Additional file 3: Figure S3.** Venogram (digital subtraction angiography) confirming the diagnosis of venous leakage.

**Additional file 4: Figure S4.** Embolization of venous leak.

## Data Availability

Patient data and materials are available.

## Abbreviations

PDE-5: Phosphodiesterase-5
IIEF: International Index of Erectile Function

## References

[CR1] Doppalapudi SK, Wajswol E, Shukla PA, Kolber MK, Singh MK, Kumar A (2019). Endovascular therapy for vasculogenic erectile dysfunction: a systematic review and meta-analysis of arterial and venous therapies. J Vasc Interv Radiol.

[CR2] Fernandez Arjona M, Oteros R, Zarca M, Diaz Fernandez J, Cortes I (2001). Percutaneous embolization for erectile dysfunction due to venous leakage: prognostic factors for a good therapeutic result. Eur Urol.

[CR3] Katzenwadel A, Popken G, Wetterauer U (1993). Penile venous surgery for cavernosal venous leakage: long-term results and retrospective studies. Urol Int.

[CR4] Kutlu R, Soylu A (2009). Deep dorsal vein embolization with N-butyl-2-cyanoacrylate and lipiodol mixture in venogenic erectile dysfunction: early and late results. Radiol Oncol.

[CR5] Peskircioglu L, Tekin I, Boyvat F, Karabulut A, Ozkardes H (2000). Embolization of the deep dorsal vein for the treatment of erectile impotence due to veno-occlusive dysfunction. J Urol.

[CR6] Rebonato A, Auci A, Sanguinetti F, Maiettini D, Rossi M, Brunese L, Carrafiello G, Torri T (2014). Embolization of the periprostatic venous plexus for erectile dysfunction resulting from venous leakage. J Vasc Interv Radiol.

[CR7] Spiliopoulos S, Shaida N, Katsanos K, Krokidis M (2013). The role of interventional radiology in the diagnosis and management of male impotence. Cardiovasc Intervent Radiol.

[CR8] Varela CG, Yeguas LAM, Rodríguez IC, Vila MDD (2020). Penile Doppler ultrasound for erectile dysfunction: technique and interpretation. AJR Am J Roentgenol.

